# Recent Evidence Regarding Triclosan and Cancer Risk

**DOI:** 10.3390/ijerph110202209

**Published:** 2014-02-21

**Authors:** Michael T. Dinwiddie, Paul D. Terry, Jiangang Chen

**Affiliations:** 1 Department of Public Health, University of Tennessee, Knoxville, TN 37996, USA; E-Mails: mdinwidd@utk.edu (M.T.D.); jchen38@utk.edu (J.C.); 2 Department of Surgery, University of Tennessee Medical Center, Knoxville, TN 37920, USA

**Keywords:** triclosan, epidemiologic studies, breast neoplasms, cancer, xenoestrogens, fatty acid synthesis

## Abstract

Triclosan is a broad-spectrum antibacterial commonly used in cosmetics, dentifrices, and other consumer products. The compound’s widespread use in consumer products and its detection in breast milk, urine, and serum have raised concerns regarding its potential association with various human health outcomes. Recent evidence suggests that triclosan may play a role in cancer development, perhaps through its estrogenicity or ability to inhibit fatty acid synthesis. Our aims here are to review studies of human exposure levels, to evaluate the results of studies examining the effects of triclosan on cancer development, and to suggest possible directions for future research.

## 1. Introduction

**Figure 1:**
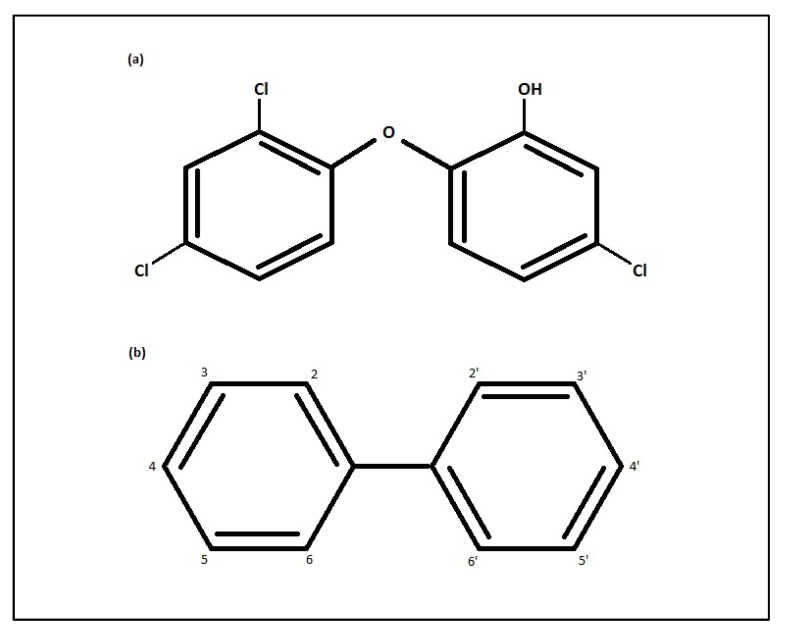
Chemical Structures of: (**a**) Triclosan and (**b**) Polychlorinated biphenyl.

Xenoestrogens are estrogen-mimicking compounds that are commonly found in personal care products, pesticides, and plastic bottles [1]. The activity of xenoestrogens in the human body involves interference with estrogen binding to estrogen receptors [1], which has implications for estrogen-dependent health outcomes including puberty, reproductive health, and pregnancy [1,2]. Xenoestrogens have attracted considerable attention in recent years as potential risk factors for cancer and other outcomes [3], which has led to some of these compounds, such as bisphenol A (BPA) and polychlorinated biphenyls (PCBs), being banned from production or from use in specific products, such as baby bottles [3]. Triclosan (5-chloro-2-(2,4-dichlorophenoxy)phenol, Figure 1a), a lesser-known xenoestrogen, is a broad-spectrum antibacterial commonly used in cosmetics, dentifrices, soap, and other consumer products [4]. The widespread use of triclosan and its detection in human breast milk, urine, and serum have raised concerns regarding its association with various health outcomes, including cancer development [4]. Our aims here are to review studies of human exposure levels, to evaluate the results of studies examining the effects of triclosan on cancer development, and to suggest possible directions for future research. 

## 2. Methods

All articles discussed in this review were received through searches of the US National Library of Medicine PubMed database or Google Scholar, which were cross-matched with the cited references of all retrieved articles. A summary of data for these studies can be found in Table 1 and Table 2. 

## 3. Results

### 3.1. Triclosan Measurement and Estimates of Human Exposure

Human exposure to triclosan occurs primarily through use of personal care products, such as toothpastes, deodorants, and soaps [5,6]. Use of these products, which typically contain 0.1 to 0.3% of the compound, results in absorption through mucosa of the gastrointestinal tract and mouth, and through the skin [5,6,7]. Following absorption, triclosan appears to be non-persistent, as free triclosan and its conjugates are rapidly eliminated within 24 hours [7]. However, several studies have found triclosan in urine, serum, and breast milk (Table 1) [7,8,9,10,11,12,13]. Calafat *et al.* measured a wide range (2.4–3,790 μg/L) of triclosan in 74.6% of 2,517 urine samples obtained from NHANES 2003–2004 [11]. Moreover, levels of triclosan in breast milk may be increased by underarm cosmetic use, which presents a direct dermal route of exposure to underlying epithelial tissue [4]. Allmyr *et al.* (2006) observed that Swedish women who are users of personal care products containing triclosan tend to have higher concentrations in milk and serum than women who use similar personal care products that presumably contain no triclosan [12]. These findings suggest the possibility that body burden can be influenced by an individual’s use of triclosan-containing products [12]. One human exposure study found that serum concentrations were as much as twofold higher in Australia than in Sweden, where consumer use of triclosan is discouraged [9]. Therefore, higher concentrations detected in China, the US, and Australia may be due to geographic differences in triclosan-containing product use. The detection of triclosan in women who reported no use of triclosan-containing consumer products suggests background exposure through other, unknown pathways. For example, it is unknown to what extent exposure to aquatic media (surface water, drinking water) contributes to concentrations of triclosan in the human body [6,14]. Nevertheless, exposure to triclosan is widespread, and despite its non-persistence, the regular use of products that contain triclosan appears to contribute to concentrations detected in humans. 

Measurement of triclosan in an individual’s urine, serum, or milk within 24 hours of exposure is likely to be a more accurate reading of the acute, but not necessarily long-term, exposure due to its non-persistence [15,16]. Unlike other lipophilic persistent xenoestrogens, such as PCBs, triclosan does not appear to accumulate in human tissue, suggesting that tissue samples may not be an adequate indicator of long-term exposure [17,18]. The dissimilarity between PCB and triclosan accumulation in tissue may be due to triclosan’s substitution of a hydroxyl group (Figure 1), which may affect its conjugation to quickly excreted water-soluble metabolites [9]. Therefore, assessment of exposure to triclosan may require other methods, such as regular scrutiny of personal care product use through questionnaires, for example, and regular measurement of exposure biomarkers, such as urine [19]. Regarding questionnaires, estimates of exposure through the use of triclosan-containing consumer products may be validated against urine levels [15], but accurate ranking of individuals in epidemiologic studies also depends, at least in part, on background exposure through other (environmental) pathways [20].

**Table 1:** Concentrations of triclosan Detected in the Environment.

Medium	Concentrations Observed	Location	Reference
Surface Water	0.0002–0.478 μg/L	US, Europe, Asia	Bedoux *et al.*, [14]
Drinking Water	0.0002–0.0145 μg/L	US, Europe, Asia	Bedoux *et al.*, [14]
WW Influent	0.052–86.2 μg/L Influent	US, Europe, Asia	Bedoux *et al.*, [14]
WW Effluent	0.028–5.37 μg/L Effluent	US, Europe, Asia	Bedoux *et al.*, [14]
Biosolids	461–30,000 ng/kg	US, Europe, Australia	Dann and Hontela, [6];Bedoux *et al.*, [14]
Serum	0.01–354 μg/L	Australia, Sweden	Allmyr *et al.*, [9]; Allmyr *et al.*, [12]
Urine	2.4–3,790 μg/L	US	Calafat *et al.*, [11]
Breast Milk	<0.018–73 μg/L	US, Australia, Sweden	Allmyr *et al.*, [9]; Adolfsson-Erici *et al.*, [10]

### 3.2. Studies of Estrogenicity and Cancer in Vitro

To date, three studies have investigated triclosan’s estrogenic action in cultured cancer cells [21,22,23]. Each of these studies utilized estrogen-receptor positive ovarian cancer (BG1Luc4E_2_) or breast cancer (MCF-7) cells exposed to triclosan, 17β-estradiol, or both [21,22,23]. Two studies examining receptor binding observed displacement of estradiol by triclosan from the ligand-binding domain of receptors, indicating that triclosan could bind to estrogen receptors in cancer cells [21,22]. All three studies observed estrogen antagonist activity by triclosan when co-exposure with estradiol resulted in markedly reduced estrogen receptor mediated gene induction and/or cell proliferation rates [21,22,23] (Table 2). Whereas antiestrogenic activity was observed in each study when estradiol was present, two studies observed proliferation in cells when triclosan was the only exposure [22,23]. The results of these studies suggest that triclosan may induce proliferation but also inhibit cell proliferation in the presence of estradiol. Increased exposure to estradiol is considered a risk factor for breast cancer development [2,21,22,23]. 

Triclosan’s intrinsic estrogenic behavior may or may not implicate the chemical as a risk factor for estrogen dependent cancers. Estrogen-dependent cancers, such as breast cancer, are known to be highly responsive to estrogens for growth. Therefore, it has been hypothesized that repeated exposure of xenoestrogens, such as triclosan, to underlying breast tissue may be a risk factor [4]. Triclosan’s estrogenicity has been previously examined in animal studies that observed increases in hepatic vitellogenin levels [6,24]. Furthermore, Gee *et al.*’s observations of receptor binding and cell proliferation add confirmation that triclosan is intrinsically estrogenic at concentrations consistent with those detected in humans (Table 1) [22]. Triclosan is similar to the xenoestrogens bisphenol A, parabens, 4-nonylphenol, and polychlorinated biphenyls in that it is, like these others, able to bind to estrogen receptors and induce proliferation in cultured estrogen-sensitive breast cancer cells [4,25]. However, triclosan’s ability to behave as an estrogen antagonist also suggests that its presence in the body alongside estradiol may actually lower risk for cancer development [22,23]. However, whether triclosan raises or lowers risk of cancer through estrogen-related pathways, and possible effect modification by estradiol, have not been examined in human studies. 

**Table 2:** Studies of Triclosan and Cancer.

Study	Concentrations Utilized	Effect Observed
Ahn *et al.*, [21]	0.0028–28.9 μg/mL	Estradiol Antagonism
Gee *et al.*, [22]	0.00002–28.9 μg/mL	Cell Proliferation, Estradiol Antagonism
Henry and Fair, [23]	0.002–200 μg/mL	Cell Proliferation, Estradiol Antagonism, Cytotoxicity
Liu *et al.*, [26]	0–20 μg/mL	FAS Inhibition, Reduced Cell Viability
Deepa *et al.*, [27] Deepa *et al.*, [28] Vandhana *et al.*, [29]	0–100 μg/mL	FAS Inhibition Reduced Cell Viability Non-Toxic to Normal Cells
Lu and Archer, [30]	1,000 ppm in diet	Reduced Mammary Tumor Incidence

### 3.3. Studies of Fatty Acid Synthesis Inhibition and Cancer in Vitro

Triclosan’s ability to inhibit fatty acid synthesis in cancer cells has been the subject of at least four studies since 2002 [31]. Fatty acid synthesis (FAS), involved in formation of phospholipid membranes and energy production, is overexpressed in several cancers including those of the breast, lung, and pancreas [31,32]. Liu and associates first observed growth inhibition in breast cancer cells following exposure of 2.5–20 μg/mL triclosan over four days, suggesting that FAS inhibition may have therapeutic potential [26]. Three recent articles have examined triclosan’s effect on inhibiting development of ocular cancer [27,28,29]. Triclosan was observed being cytotoxic to Y79 retinoblastoma cells in a dose and time-dependent manner following exposure of concentrations up to 100 μg/mL [27]. Furthermore, normal human MIOM1 ocular and 3T3 mouse fibroblast cells were unaffected by the IC_50 _of triclosan in cancer cells, indicating a high therapeutic index (TI) for triclosan [27,31]. This indicates that triclosan’s FAS inhibiting mechanism may be especially harmful to cancer cells and therefore may present little danger to surrounding normal cells [27,28,29]. Together, the results of these *in vitro* studies suggest that FAS inhibitors, such as triclosan, may be an effective inhibitor of cancer growth, though no study has examined whether triclosan reduces cancer growth or incidence in humans, or what co-factors may potentiate any effect. 

Liu *et al.* observed reduced cell growth and viability at a range of 2.5–20 μg/mL [26]. In contrast, Henry and Fair observed increased cell proliferation or estradiol antagonism by triclosan at a very similar range [23]. Although the reason for the discrepancy is unclear, study length may have influenced the results of these studies. Henry and Fair’s results were based on only one day of exposure, whereas Liu *et al.* observed reduced viability and a decreasing IC_50_ in MCF-7 cells across four days [23,26]. This decrease in IC_50_ has also been observed in the studies examining retinoblastoma cells over 96 hours [27,28,29]. Furthermore, Henry and Fair reported slight cytotoxic responses beginning at ~20 μg/mL, within the range observed by both Liu *et al.* and Deepa *et al.* (2012) [26,27]. Whereas triclosan exposure (up to 20 μg/mL) may induce proliferation or antagonize estradiol, the duration of exposure may be positively associated with cytotoxicity [23]. Henry and Fair speculated that environmentally relevant exposures would elicit greater toxic responses across extended exposure periods [23]. 

### 3.4. Studies of Triclosan in Animal Experiments

Animal studies of triclosan exposure and cancer development have included long-term studies of its carcinogenicity and short-term studies examining fatty acid synthase-related cancer growth inhibition [15,30]. Previously, chronic animal studies on rats, hamsters, and baboons observed no increase in carcinogenesis [15]. However, one study did observe the appearance of liver tumors in mice following 18 months of exposure to 0–200 mg/kg/day triclosan in diet. These tumors were produced through peroxisome proliferator-activated receptor α (PPARα), though this mechanism was deemed not applicable to humans as PPARα agonists have not been shown to increase carcinogenesis in the livers of humans [15]. In contrast, Lu and Archer [30] found that tumor development was inhibited by triclosan exposure. Following injection with 50 mg/kg of methylnitrosourea, a carcinogen, Sprague-Dawley rats fed a diet supplemented with 1,000 ppm triclosan (which may increase blood serum concentrations up to 86.7 μg/mL) showed a markedly reduced incidence of mammary tumors than rats fed a non-supplemented diet [5,30]. Studies on algae and zebra mussels suggest that triclosan may be genotoxic at high concentrations, though this effect has not been observed in studies of mammal cell lines exposed to environmentally-relevant levels of triclosan [6]. Overall, however, the results of animal studies to date have been mixed, showing null, inverse, and positive associations. 

### 3.5. Triclosan and Cancer in Human Studies

Investigators have yet to translate the suggestive findings of *in vitro* and animal studies to human health in the published literature. Rather, human studies involving triclosan have primarily focused on the chemical’s safety, pharmacokinetics, and antimicrobial effectiveness [6,15]. Both short and long-term studies have examined triclosan following oral and dermal exposure, showing rapid excretion from the body and no evidence of toxicity, irritation, or thyroid hormone disruption [6,15]. 

Whereas previous human studies have not clearly implicated triclosan in any health condition, they also have not addressed experimental findings that suggest triclosan’s estrogenicity may function to either stimulate or inhibit human estrogen-dependent cancer cell growth. Neither have subsequent published studies addressed the results of *in vitro* and animal studies suggesting triclosan may inhibit growth of FAS expressing cancers [26,27,28,29]. The translation of findings from *in vitro* studies and controlled animal experiments to the examination of health effects in free living humans is challenging, but a reasonable first step may be a study in which cancer cases and controls are investigated for their prior exposure to triclosan through product use or occupational exposure. Longitudinal studies of cancer patients that include repeated measures of triclosan exposure might also help to clarify any association with neoplastic growth. 

## 4. Conclusions

Recent evidence suggests that triclosan exposure may alter cancer risk, although human studies are lacking in both number and scope. Therefore, epidemiologic studies of risk associated with various concentrations and durations of exposure to triclosan are needed, as well as studies to characterize human exposure to triclosan through varying use of triclosan-containing consumer products and other routes of exposure. 

## References

[1] Roy J.R., Chakraborty S., Chakraborty T.R. (2009). Estrogen-like endocrine disrupting chemicals affecting puberty in humans—A review. Med. Sci. Monit..

[2] Fernandez S.V., Russo J. (2010). Estrogen and xenoestrogens in breast cancer. Toxicol. Pathol..

[3] Erler C., Novak J. (2010). Bisphenol a exposure: Human risk and health policy. J. Pediatr. Nurs..

[4] Darbre P.D. (2006). Environmental oestrogens, cosmetics and breast cancer. Best Pract. Res. Clin. Endocrinol. Metab..

[5] Bhargava H.N., Leonard P.A. (1996). Triclosan: Applications and safety. Am. J. Infect. Control.

[6] Dann A.B., Hontela A. (2011). Triclosan: Environmental exposure, toxicity and mechanisms of action. J. Appl. Toxicol..

[7] Sandborgh-Englund G., Adolfsson-Erici M., Odham G., Ekstrand J. (2006). Pharmacokinetics of triclosan following oral ingestion in humans. J. Toxicol. Environ. health. Part A.

[8] Moss T., Howes D., Williams F.M. (2000). Percutaneous penetration and dermal metabolism of triclosan (2,4,4'-trichloro-2'-hydroxydiphenyl ether). Food Chem. Toxicol..

[9] Allmyr M., Harden F., Toms L.M., Mueller J.F., McLachlan M.S., Adolfsson-Erici M., Sandborgh-Englund G. (2008). The influence of age and gender on triclosan concentrations in Australian human blood serum. Sci. Total Environ..

[10] Adolfsson-Erici M., Pettersson M., Parkkonen J., Sturve J. (2002). Triclosan, a commonly used bactericide found in human milk and in the aquatic environment in Sweden. Chemosphere.

[11] Calafat A.M., Ye X., Wong L.Y., Reidy J.A., Needham L.L. (2008). Urinary concentrations of triclosan in the U.S. Population: 2003–2004. Environ. Health Perspect..

[12] Allmyr M., Adolfsson-Erici M., McLachlan M.S., Sandborgh-Englund G. (2006). Triclosan in plasma and milk from swedish nursing mothers and their exposure via personal care products. Sci. Total Environ..

[13] Dayan A.D. (2007). Risk assessment of triclosan [irgasan] in human breast milk. Food Chem. Toxicol..

[14] Bedoux G., Roig B., Thomas O., Dupont V., Le Bot B. (2012). Occurrence and toxicity of antimicrobial triclosan and by-products in the environment. Environ. Sci. Pollut. Res. Int..

[15] Rodricks J.V., Swenberg J.A., Borzelleca J.F., Maronpot R.R., Shipp A.M. (2010). Triclosan: A critical review of the experimental data and development of margins of safety for consumer products. Crit. Rev. Toxicol..

[16] Laas E., Poilroux C., Bezu C., Coutant C., Uzan S., Rouzier R., Chereau E. (2012). Antibacterial-coated suture in reducing surgical site infection in breast surgery: A prospective study. Int. J. Breast Cancer.

[17] Toms L.M., Allmyr M., Mueller J.F., Adolfsson-Erici M., McLachlan M., Murby J., Harden F.A. (2011). Triclosan in individual human milk samples from australia. Chemosphere.

[18] Geens T., Neels H., Covaci A. (2012). Distribution of bisphenol-a, triclosan and n-nonylphenol in human adipose tissue, liver and brain. Chemosphere.

[19] Calafat A.M., Ye X., Silva M.J., Kuklenyik Z., Needham L.L. (2006). Human exposure assessment to environmental chemicals using biomonitoring. Int. J. Androl..

[20] Calafat A.M., M. K.H., Swan S.H., Hauser R., Goldman L.R., Lanphear B.P., Longnecker M.P., Rudel R.A., Teitelbaum S.L., Whyatt R.M., Wolff M.S. (2013). Misuse of blood serum to assess exposure to bisphenol a and phthalates. Breast Cancer Res..

[21] Ahn K.C., Zhao B., Chen J., Cherednichenko G., Sanmarti E., Denison M.S., Lasley B., Pessah I.N., Kultz D., Chang D.P., Gee S.J., Hammock B.D. (2008). *In vitro* biologic activities of the antimicrobials triclocarban, its analogs, and triclosan in bioassay screens: Receptor-based bioassay screens. Environ. Health Perspect..

[22] Gee R.H., Charles A., Taylor N., Darbre P.D. (2008). Oestrogenic and androgenic activity of triclosan in breast cancer cells. J. Appl. Toxicol..

[23] Henry N.D., Fair P.A. (2013). Comparison of *in vitro* cytotoxicity, estrogenicity and anti-estrogenicity of triclosan, perfluorooctane sulfonate and perfluorooctanoic acid. J. appl. Toxicol..

[24] Stoker T.E., Gibson E.K., Zorrilla L.M. (2010). Triclosan exposure modulates estrogen-dependent responses in the female wistar rat. Toxicol. Sci..

[25] Recchia A.G., Vivacqua A., Gabriele S., Carpino A., Fasanella G., Rago V., Bonofiglio D., Maggiolini M. (2004). Xenoestrogens and the induction of proliferative effects in breast cancer cells via direct activation of oestrogen receptor alpha. Food add. Contam..

[26] Liu B., Wang Y., Fillgrove K.L., Anderson V.E. (2002). Triclosan inhibits enoyl-reductase of type I fatty acid synthase *in vitro* and is cytotoxic to mcf-7 and skbr-3 breast cancer cells. Cancer Chemother Pharm..

[27] Deepa P.R., Vandhana S., Muthukumaran S., Umashankar V., Jayanthi U., Krishnakumar S. (2010). Chemical inhibition of fatty acid synthase: Molecular docking analysis and biochemical validation in ocular cancer cells. J. Ocul. Boil. Dis. Informa..

[28] Deepa P.R., Vandhana S., Jayanthi U., Krishnakumar S. (2012). Therapeutic and toxicologic evaluation of anti-lipogenic agents in cancer cells compared with non-neoplastic cells. Basic Clin. Pharm. Toxicol..

[29] Vandhana S., Coral K., Jayanthi U., Deepa P.R., Krishnakumar S. (2013). Biochemical changes accompanying apoptotic cell death in retinoblastoma cancer cells treated with lipogenic enzyme inhibitors. Biochimica et Biophysica Acta.

[30] Lu S., Archer M.C. (2005). Fatty acid synthase is a potential molecular target for the chemoprevention of breast cancer. Carcinogenesis.

[31] Kuhajda F.P. (2000). Fatty-acid synthase and human cancer: New perspectives on its role in tumor biology. Nutrition.

[32] Flavin R., Peluso S., Nguyen P.L., Loda M. (2010). Fatty acid synthase as a potential therapeutic target in cancer. Future Oncol..

